# Digitized capillary surface profile tables from Bashforth and Adams, 1883

**DOI:** 10.1038/s41597-026-07917-6

**Published:** 2026-07-21

**Authors:** Donna Post Guillen, Daniel Yankura, Michael Wu

**Affiliations:** Idaho National Laboratory, Idaho Falls, Idaho 83415 USA

## Abstract

Bashforth and Adams (1883) published solutions to the Young–Laplace equation governing axisymmetric capillary surfaces, providing tabulated dimensionless profile coordinates (x/b and z/b) as functions of the angle (Φ) and the dimensionless capillary parameter (β) for sessile drops, pendant drops, and capillary menisci. These tables have remained the authoritative reference for surface tension analysis by the axisymmetric drop shape method for over 140 years. The original monograph is out of print and surviving library copies are of poor scanned quality, making accurate extraction of the tabulated values laborious and error-prone. This article presents a machine-readable digitization of Table II (pp. 4–23) from the original work. Table II provides 100 values of β ranging from 0.125 to 100, with 36 Φ increments per β value (5° to 180° in 5° steps), yielding x/b and z/b coordinate pairs. Data were manually transcribed from scanned images of the original tables, organized by source page in separate worksheets of a single Microsoft Excel workbook, and validated through visual quality assurance (QA) plots of the profile curves on each worksheet. The dataset is freely available to support surface tension research and to eliminate the need for researchers to independently re-digitize this widely used historical resource.

## Background & Summary

The shape of a static axisymmetric liquid surface is governed by the Young–Laplace equation, which equates the Laplace pressure arising from surface curvature to the hydrostatic pressure difference across the interface^1,2^. For a drop or meniscus with an axis of symmetry, this equation can be written in dimensionless form as a system of ordinary differential equations parameterized by the arc length along the meridional profile. This formulation collapses to a single dimensionless group, the capillary parameter β = bΔρg/γ, combining the apex radius of curvature (b), the density contrast between the two fluid phases (Δρ), the local gravitational acceleration (g), and the interfacial tension (γ). The resulting profile is described by the dimensionless coordinates x/b (radial distance from the axis) and z/b (axial distance measured downward from the apex).

Francis Bashforth and John Couch Adams took on this problem in 1883, publishing “An Attempt to Test the Theories of Capillary Action” after numerically integrating the governing equation across a broad span of β values and compiling the resulting coordinates into tabular form^3^. Given the hand calculation and finite-difference methods available at the time, the effort represented a considerable computational feat. Table II went on to become the field’s standard reference for the axisymmetric drop shape method.

The axisymmetric drop shape analysis (ADSA) technique, formalized in the 1980s and 1990s, fits the theoretical Young–Laplace profile to the digitized outline of an experimental drop image to extract surface tension^4,5^. ADSA implementations rely either on direct interpolation of the Bashforth–Adams tables or on numerical integration of the governing equations at runtime. The tabulated values also appear in instructional and reference contexts, as they provide physical intuition about how drop shape depends on the ratio of surface tension to gravitational forces.

Despite the importance of this dataset, no clean machine-readable version has been widely distributed. Researchers who need the values have historically re-typed them from physical or scanned copies of the monograph, or derived them independently by numerical integration. The original monograph is out of print and surviving copies exist only as low-quality scans; any research group requiring the tabulated values has faced the same painstaking manual transcription task, susceptible to error. Having completed this work for our own surface tension analyses, we believe it is of clear community value to make the digitized dataset openly available so that others need not repeat the effort.

The dataset directly supports reproducible research in surface science, physical chemistry, food science, petroleum engineering, pharmaceutical science, and any other field in which ADSA-based surface tension measurement is used.

## Methods

### Source material

The source document is: Bashforth, F. and Adams, J.C. (1883). An Attempt to Test the Theories of Capillary Action. Cambridge University Press, Cambridge, UK^3^. The monograph is commonly miscited with a publication date of 1892, which corresponds to the death year of co-author John Couch Adams as it appears in library catalogue records (Adams, John Couch, 1819–1892); the correct and only edition was published in 1883. A scanned PDF copy of the monograph was obtained from a university library. The scan resolution and image quality varied across pages, with some pages exhibiting significant degradation. No other editions or printings of the original tables were available for cross-reference.

### Digitization procedure

Table II was identified as the primary results table of the monograph. The following procedure was applied to each page:

- The relevant pages of the scanned monograph were enlarged and examined at sufficient zoom to read individual digits.
- Data were entered manually into the Excel workbook, one page of the original table per worksheet, preserving the original column arrangement of five β values per page.
- After completing each worksheet, a QA scatter plot of z/b vs x/b was generated in Excel with one data series per β value. Any data point deviating conspicuously from the expected smooth, monotonically varying curve was reviewed and corrected if a transcription error was identified.
- Numeric cell formatting was set to five decimal places to preserve the printed significant figures and facilitate visual inspection.
- No automated or optical character recognition (OCR) tools were used; all values were entered and checked manually. In total, 7,200 (x/b, z/b) values were transcribed (100 β values × 36 Φ increments × 2 coordinates).

### Workbook organization

The Excel workbook contains 21 worksheets. The “QA Notes” worksheet documents the source file name, scope of digitization, display precision conventions, and the QA plot convention. The remaining 20 worksheets (“Table II_p4” through “Table II_p23”) each contain data from one printed page of Table II. Sheet names encode the source page number. Within each sheet, the first row contains the β values for that page, the second row contains the column headers (x/b and z/b alternating), and subsequent rows contain the data indexed by Φ.

## Data Records

The dataset is provided as a single Microsoft Excel workbook (.xlsx) deposited at the Nuclear Research Data System (NRDS), https://doi.org/10.48806/3365240^6^. The workbook contains 21 worksheets organized by source page of the original monograph.

### Table II — dimensionless drop profile coordinates

Table II of Bashforth and Adams (1883) tabulates, for each of 100 values of β (ranging from 0.125 to 100), the dimensionless radial coordinate x/b and the dimensionless axial coordinate z/b at 36 values of the angle Φ from 5° to 180° in increments of 5°. The angle Φ is the angle which the normal to the surface makes with the axis of revolution. Together, Φ, x/b, and z/b parameterize the axisymmetric capillary surface from apex to contact line.

The β values are not uniformly spaced across the full range. For small β (0.125 to 0.25), the increment is 0.125; from β = 0.25 to 1 the increment is 0.25; for β = 1 to 20 the increment is 0.5; from β = 20 to 50 the increment is 1; from β = 50 to 96 the increment is 2; and from β = 96 to 100 the increment returns to 1. This non-uniform spacing reflects the practical range of drop shapes encountered in experimental measurements and the varying sensitivity of profile shape to β in different regimes.

### Variable definitions

The variables used in the tables are defined in accordance with the original Bashforth–Adams notation and standard ADSA practice:

- β — Dimensionless capillary parameter, β = bΔρg/γ, expressed as a function of the apex radius of curvature (b), the liquid–gas density difference (Δρ), gravitational acceleration (g), and the interfacial tension (γ). Large β corresponds to strong gravitational distortion; small β to nearly spherical shapes.
- b — Radius of curvature at the drop apex; serves as the length scale for nondimensionalization.
- x/b — Dimensionless radial coordinate. The horizontal distance from the axis of symmetry to a point on the drop profile, nondimensionalized by b.
- z/b — Dimensionless axial (vertical) coordinate. The vertical distance measured downward from the apex to a point on the drop profile, nondimensionalized by b.
- Φ — Angle (degrees). The angle which the normal to the surface makes with the axis of revolution. Ranges from 5° (near apex) to 180° (equatorial plane or contact line) in Table II.

**Fig. 1:**
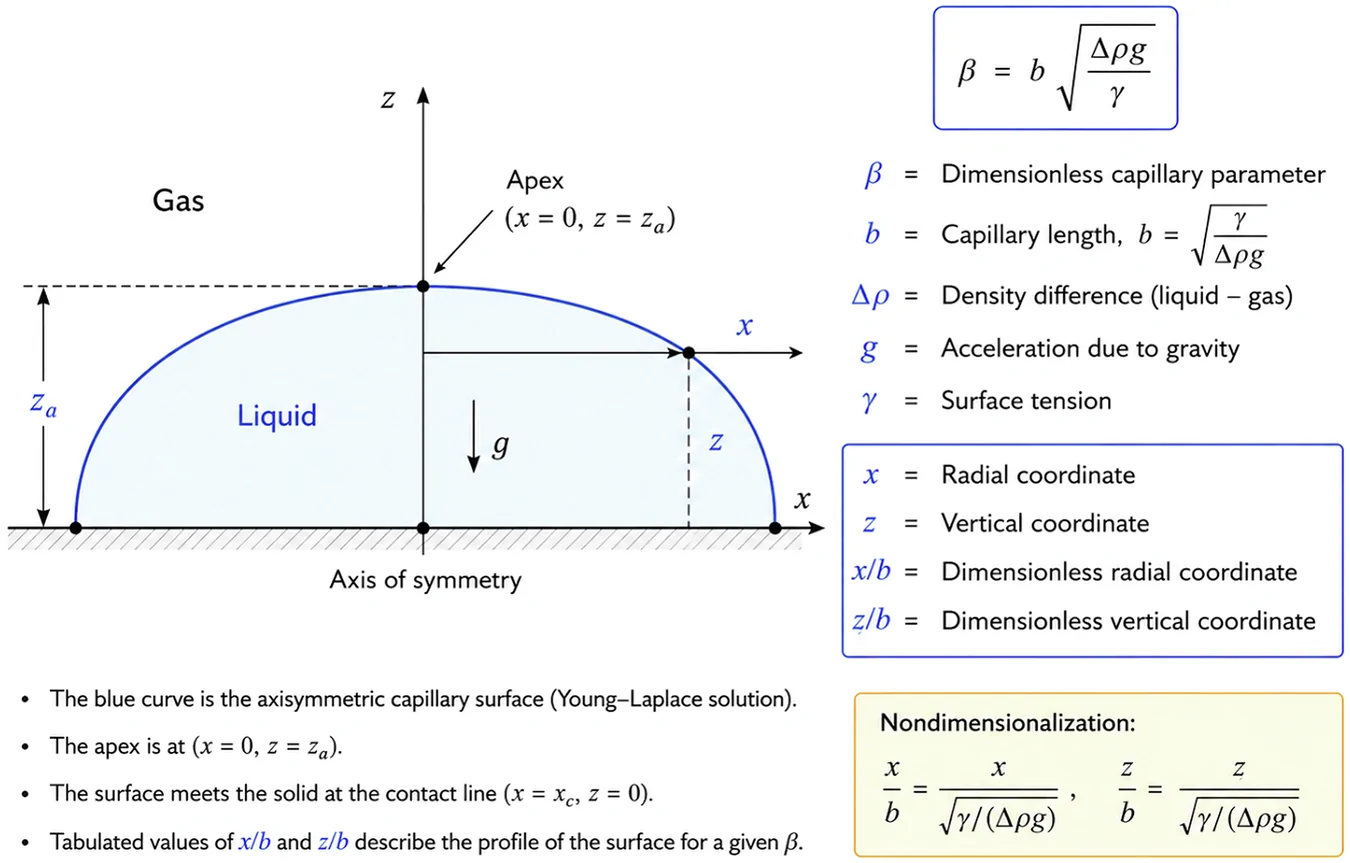
Definition of variables for a sessile drop.

The Bashforth–Adams procedure assumes that the drop is symmetric about the z-axis. These definitions are consistent with those illustrated in Fig. 1.

## Technical Validation

Data quality assurance was performed visually. After completing each worksheet, a scatter plot of z/b vs x/b was generated in Excel with one data series per β value. The Young–Laplace solutions produce a family of smooth, monotonically varying profile curves; any transcribed data point deviating conspicuously from this expected behavior was flagged, reviewed against the scanned source, and corrected if a transcription error was identified. The embedded QA plots remain in each worksheet, enabling readers to perform the same check independently.

A number of entries in the original tables were difficult to read due to scan quality, typographical quirks of the 1883 typesetting, or ink bleed. In all such cases, the best-effort value was entered. Occasional minor typographical irregularities were observed in the original tables, consistent with hand-composition printing of that era (e.g., a digit readable as either of two similar characters, or a value slightly out of sequence in what should be a monotone progression).

The QA plots provide a qualitative rather than quantitative accuracy check: they are effective at detecting gross transcription errors (wrong digit, transposed digits, missed decimal point) but would not reliably identify errors of one unit in the last printed digit. The original tables were computed with finite-difference methods and are themselves subject to the numerical precision of 1883-era hand calculation; the digitized values faithfully reproduce the printed values but do not correct for any original computational errors.

The primary limitation is that digitization relied on a single, degraded scanned copy of the original monograph. No independent verification against a second physical copy or against a numerical re-integration of the Young–Laplace equation was performed. The column header notation in the original uses “z/b” for the axial coordinate; some historical references use the symbol ζ or c for this variable. The notation in this dataset follows the original Bashforth–Adams convention.

## Usage Notes

The workbook preserves the original page structure of the monograph to facilitate verification against the source material. Users requiring a consolidated flat table can readily combine the 20 Table II worksheets using standard spreadsheet operations (e.g., Power Query in Excel) or scripting (e.g., Python with openpyxl or pandas).

Researchers requiring high-confidence values for specific β and Φ combinations are encouraged to cross-check against a numerical integration of the Young–Laplace equation, for which several open-source implementations exist. ADSA software packages that perform their own numerical integration can use these digitized tables as a validation reference.

### Ethics statement

No human subjects, animals, or personal data were involved in the production of this dataset. The source material (Bashforth and Adams, 1883) is in the public domain.

## Supplementary information

Supplementary Information

## Data Availability

The digitized dataset is available at the Nuclear Research Data System (NRDS): https://doi.org/10.48806/3365240^6^.

## Code availability

No custom code was used in the production of this dataset. All digitization was performed manually using Microsoft Excel.
